# Smoothed Growth Trajectories for Weight, Length/Height, and BMI Percentiles of Children Aged 3 to 60 Months in Punjab, Pakistan

**DOI:** 10.3390/jcm14061949

**Published:** 2025-03-13

**Authors:** Natasha Akbar, Muhammad Aslam, Syed Muhammad Dure Sabih Haider, Muhammad Asif, Piotr Matłosz, Justyna Wyszyńska

**Affiliations:** 1 Department of Statistics, Bahauddin Zakariya University, Multan 60800, Pakistan; natashaakbar86@gmail.com; 2 Department of General Pediatric Medicine, The Children Hospital and Institute of Child Health, Multan 60000, Pakistan; sabihhaider39@gmail.com; 3 Department of Statistics, Government Graduate College, Qadir Pur Raan, Multan, Pakistan; asifmalik722@gmail.com; 4 Faculty of Physical Culture Sciences, Collegium Medicum, University of Rzeszow, 35-959 Rzeszów, Poland; 5 Faculty of Health Sciences and Psychology, Collegium Medicum, University of Rzeszow, 35-959 Rzeszów, Poland; jwyszynska@ur.edu.pl

**Keywords:** anthropometric measurements, body mass index, Box–Cox power exponential distribution, growth references chart, Lambda–mu–sigma, smoothed percentiles

## Abstract

**Background/Objectives**: This study aims to examine the crucial role of children’s growth in assessing population health trends and developing targeted interventions. Specifically, the research aims to determine prevalent anthropometric trajectories among children from Punjab, Pakistan, and to compare these patterns with global and national growth standards established by the World Health Organization (WHO). **Methods**: A cross-sectional survey was conducted to assess the weight and height of 20,845 children (51.48% boys and 48.52% girls) aged 3 to 60 months in Punjab, Pakistan. Growth reference charts for boys and girls were developed using the generalized additive model for location, scale, and shape, utilizing the Box–Cox power exponential distribution. The parameters analyzed included weight-for-age, length/height-for-age, and body mass index (BMI)-for-age. **Results**: The 3rd, 5th, 15th, 25th, 50th, 75th, 85th, 95th, and 97th smoothed percentile values with L, M, and S for weight, length/height, and BMI for both sexes from 3 to 60 months were presented. The median weight and length/height were increased significantly in both sexes. The median BMI increased steeply in early life, with a peak at 12 months, then declined in both boys and girls. Boys had a higher mean value than girls had in all the anthropometric variables. Punjabi children demonstrate smaller measurements compared to their counterparts in previous studies. The children were smaller, lighter and had lower BMI than that referred to in the WHO standards. The findings highlighted significant disparities between the growth metrics of children in Punjab and the WHO standards, and findings from other global studies. **Conclusions**: The study highlights the need for a multicentric approach in future research to better understand pediatric growth patterns in Punjabi children, considering their significant differences from WHO standards and other global studies.

## 1. Introduction

Early growth serves as a positive indicator of a community’s overarching socioeconomic trends and overall health. Infant growth is influenced by both internal factors, such as genetic potential, and external factors, including lifestyle and nutrition. Body mass index (BMI), weight, and height can reflect these fluctuating factors and broader secular trends over time. Therefore, it is essential to regularly update national growth references to account for these variations [1]. Growth charts and references play a crucial role in pediatric clinical assessments and child health evaluation [2]. The data utilized to update the World Health Organization’s (WHO) Guidelines for Child Growth and Development in 2006 were derived from a multicenter study conducted between 1997 and 2003, encompassing six participating countries: Belgium, China, India, Norway, Oman, and the United States [3]. The Pakistani government agreed to utilize the WHO 2006 growth standards for tracking the growth of children under five. Nevertheless, numerous international and national studies [4,5,6,7] indicate that these standards remain aspirational; consequently, many children have been classified as stunted and undernourished despite growing normally within their demographic group. Numerous studies in Pakistan [7,8,9,10,11,12,13] have provided growth charts and percentiles for weight, height, and BMI in children and adolescents; however, none have specifically determined the smoothed percentiles for children aged 3 to 60 months in the province of Punjab. We established the new growth reference percentiles and curves for children residing in Punjab. Currently, data from the Punjab province of Pakistan are utilized to assess the nutritional status of children. Given that anthropometric data typically follow a non-Gaussian distribution [5], the lambda–mu and sigma (LMS) method through the implementation of the generalized additive model for location scale and shape (GAMLSS) was used. In this study, we calculated smoothed empirical percentiles for boys and girls in the Punjab province regarding their weight-for-age, length/height-for-age, and BMI-for-age while taking into account skewness and kurtosis.

The objectives of this research were as follows: (I) to establish first growth references and percentiles based on anthropometric measurements of healthy children from Punjab aged 3 to 60 months; (II) to compare these new references with existing national standards and the WHO guidelines; and (III) to provide more precise and accurate references for clinical practice and scientific research for at the provincial level.

## 2. Materials and Methods

### 2.1. Sample and Design

With authorization from Pakistan’s Ministry of National Health Services, Regulations, and Coordination, the United Nations Children’s Fund (UNICEF) carried out the 2018 National Nutrition Survey (NNS) from 2018 to 2019. This survey provided the most comprehensive collection of nutritional data ever gathered for households with children under five, adolescents, and women of reproductive age in both urban and rural settings.

A total of 68,493 interviews were conducted with mothers or caregivers of children from birth to 60 months, achieving an impressive response rate of 84.20%. Mothers present during the home visit were interviewed, and those caring for young children completed a questionnaire. Some sections of the survey were specifically designed for particular age groups, such as newborns and young children who still receive breast milk at 24 months. The data focused on children and newborns in good health, as determined by their medical history and physical examinations, excluding those with confirmed chronic illness, preterm infants, or any medical conditions that could hinder their growth, including dietary deficiencies. Anthropometric measures were measured according to standardized techniques [14]. This study focuses on Punjab, Pakistan, the country’s largest province with nearly 110 million people and an area of 205,345 km^2^. Punjab is divided into 9 divisions and 36 districts, featuring diverse geography, climate, and rural–urban environments [15]. The final sample included 20,845 participants (10,731 boys and 10,144 girls) aged between 3 to 60 months, with anthropometric measurements taken for length in centimeters (cm) from 3 months to 24 months, height (cm) from 2 to 5 years, and weight in kilogram (kg).

To maintain the credibility and consistency of these findings, outliers and missing data were excluded according to criteria detailed in recent studies [7,13], thereby enhancing the robustness and reliability of the results.

### 2.2. Anthropometric Measurements

This investigation utilized anthropometric variables, including weight (kg) and height (cm). The BMI was calculated using the following formula: weight in kilograms divided by height in square meters squared. Anthropometric measurements were conducted utilizing the standardized monitoring and assessment of relief and transitions approach. Every child was weighed using a Seca 874 U electronic scale (Seca, Hamburg, Germany), with measurements taken while dressed in lightweight clothing and without shoes, to the nearest 0.10 kg. The length of children aged 3–24 months and the heights of those aged 25–60 months were recorded using a standard infantometer (Chasmors Ltd., London, UK) and stadiometer (Seca GmbH & Co., KG, Germany), respectively [14].

### 2.3. Statistical Methods and Analysis

**Figure 1:**
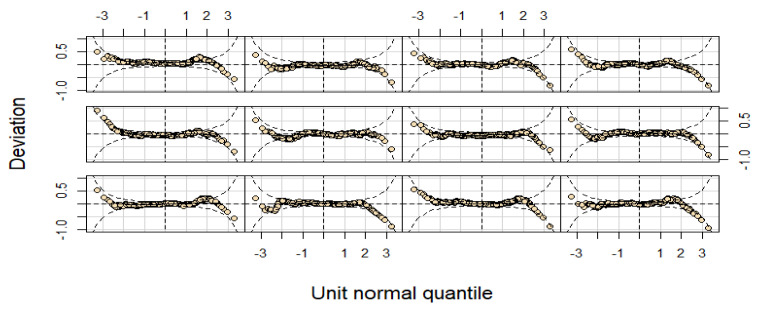
Worm plots from the BCPE model.

The analysis was conducted using R version 4.2.2 (R Foundation, Vienna, Austria). Using the LMS technique and GAMLSS (version 5.3.4) [16,17] with the Box–Cox power exponential (BCPE) distribution [18], we constructed smoothed growth reference curves and percentiles for weight-for-age, length/height-for-age, and BMI-for-age of children aged 3 to 60 months, evaluated at 3-month intervals. When anthropometric measurements, such as weight, length/height, and BMI, exhibit a Gaussian distribution, the essential criterion for centile estimation is the Z-scores represented in terms of centiles. The basic characteristics of the Gaussian distribution are as follows: the anthropometric variables’ frequency curve should be unimodal, symmetrical (skewness = 0), and have a normal peak (kurtosis = 3) [19]. The best approaches for both smoothness and normality are represented by the LMS method and its modification [18], the BCPE [20], which consists of four parameters which are represented by the symbols μ, σ, ν, and τ. The location (median), scale (approximate coefficient of variation), skewness (transformation to the symmetry), and kurtosis (power exponential parameter) were correlated with these parameters, indicating that order. This approach, often referred to as the Lambda–mu–sigma-Power (LMSP) [21] method and a generalization of the LMS method of the centile estimate, includes an additional parameter for determining the accuracy of kurtosis called P = τ. These four characteristics are modeled by the GAMLSS model using the BCPE distribution [18] as functions of several explanatory variables. Given that the only explanatory variable in the GAMLSS model is age, centile estimation is a specific case of the model. Relying on four distribution parameters (µ, σ, ν, and τ), the GAMLSS model is a semi-parametric model with a probability density function. The first two are parameters of the population distribution that describe location and scale: the mean (µ) and the corresponding coefficient of variation (σ). The remaining two are shape parameters: τ (kurtosis) and ν (skewness). Using the Rigby–Stasinopoulos (RS) technique [18], these four parameters were modeled as a non-parametric smoothing cubic spline function of age with effective degrees of freedom (EDF) and BCPE distribution. The ultimate models used for the present research were in line with another study [7,13]. For further details, see Appendix A, Table A1. Tools for residual diagnosis for all the models, i.e., quantile residuals, worm plots, and Q-Q plots, have been employed to check the normality of the residuals (Figure 1).

Due to the brevity of space, the worm plots about the weight parameters for boys only have been displayed in Figure 1. For both sexes, the models exhibiting the lowest global deviation were accepted. As a result, the age-transformation power values in the BCPE distribution ranged from 0.08 to 0.90. The model’s parameter with the lowest global deviation was chosen. The theoretical and empirical percentiles of the fitted model were compared using the previously reported approaches [7,13,15,20] to ensure that all models had an adequate fit (Appendix A, Table A1). The new reference charts and percentile values about weight-for-age, length/height-for-age, and BMI-for-age were computed for the children living in the province of Punjab.

## 3. Results

**Figure 2:**
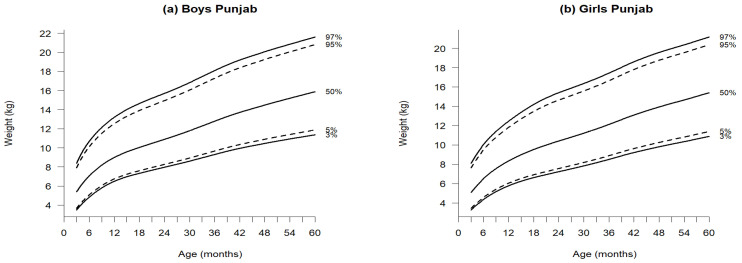
Percentiles of weight-for-age of boys (**a**) and girls (**b**) aged 3–60 months in Punjab, Pakistan.

**Figure 3:**
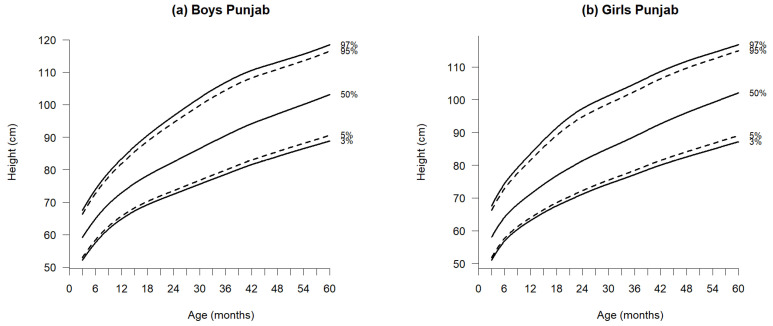
Percentiles of height-for-age of boys (**a**) and girls (**b**) aged 3–60 months in Punjab, Pakistan.

**Figure 4:**
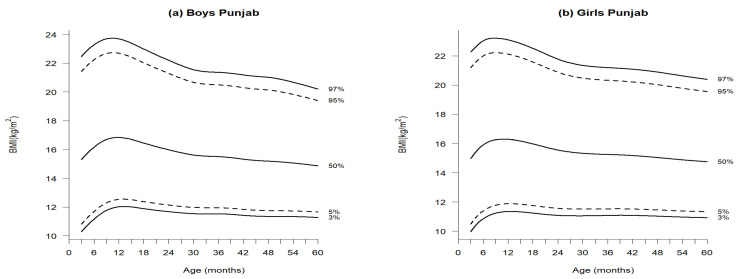
Percentiles of BMI-for-age of boys (**a**) and girls (**b**) aged 3–60 months in Punjab, Pakistan.

Figure 2, Figure 3 and Figure 4 illustrate the growth reference percentiles for anthropometric measurements, including weight-for-age, length/height-for-age, and BMI-for-age for boys and girls aged 3 to 60 months in the Punjab region, covering five percentiles P_3_, P_5_, P_50_, P_95_, and P_97_. Supporting data are provided in Appendix A—Table A2 and Table A3, which include descriptive statistics for weight-for-age, length/height-for-age, and BMI at 3-month intervals within this age range. Additionally, Table 1, Table 2 and Table 3 present the nine reference percentile values from P_3_ to P_97_, calculated using the GAMLSS method.

### 3.1. Weight-for-Age

At 3 months, the median weight was 5.38 kg for boys and 5.07 kg for girls, increasing to 15.87 kg for boys and 15.39 kg for girls by 60 months (Figure 2 and Table 1a,b). Both sexes exhibited a significant increase in weight during the first eighteen months, with boys consistently weighing more than girls by 0.31 kg to 0.48 kg across all age categories. The median weight P50 showed significant increases in both boys and girls. Notably, the nine percentiles for boys consistently exceeded those for girls. Examining the widths between P_3_ and P_97_ percentiles for weight, references indicated narrow spans from 3 to 60 months for P_3_ and P_5_. Conversely, percentiles from P_15_ to P_97_ exhibited wider ranges compared to other growth references.

In accordance with WHO standards [22], boys and girls at 3 months weighed 1.02 kg and 0.73 kg more, respectively, compared to the newly established Punjab references. This weight discrepancy widened as children aged, reaching 1.32 kg for boys and 1.1 kg for girls at 24 months, 1.52 kg for boys and 1.78 kg for girls at 36 months, and 1.83 kg for boys and 2.18 kg for girls at 48 months. The most significant difference was noted at 60 months, with boys weighing 2.43 kg and girls weighing 2.81 kg more than their counterparts in the Punjab reference. In this age group, the median weight of girls, based on WHO standards, surpassed that of the newly formulated references (Table 4).

### 3.2. Length/Height-for-Age

At 3 months, boys measured a median length of 59.15 cm, while girls measured 58.03 cm; at 60 months, the median heights for boys and girls were 103.12 cm and 102.05 cm, respectively (Figure 3 and Table 2a,b).

Throughout both time points, boys consistently outperformed girls in height, exhibiting a difference of 1.12 cm at three months and 1.07 cm at 60 months. This trend persisted across age groups, with boys consistently surpassing girls in height, while both sexes demonstrated an increase in length/height as they aged. Although boys generally presented higher percentile curves than girls, the median height and length exhibited only minor variations, ranging from 0.89 to 1.74 cm. At 12 months, boys measured an average of 1.74 cm taller than girls, a trend characterized by greater variability in this age group compared to others. When comparing WHO standards [22] to the newly established percentiles, the median difference ranged from 1.70 cm to 7.35 cm for both sexes, with a more significant disparity becoming evident after 24 months of age (Table 4).

### 3.3. BMI-for-Age

In both sexes, the median BMI percentiles were 15.31 kg/m^2^ for boys and 14.97 kg/m^2^ for girls at 3 months. By 12 months, BMI sharply increased to 16.84 kg/m^2^ for boys and 16.30 kg/m^2^ for girls, then gradually declined by 24 months to 15.98 kg/m^2^ in boys and 15.56 kg/m^2^ in girls. Despite a slight difference of 0.34 at 3 months and 0.32 at 60 months, boys consistently exhibited higher BMI than girls.

Due to the absence of published median BMI references for the initial Punjab province in Pakistan, the median BMI values from the new complete cohort were exclusively compared to international standards. The BMI values for boys at P_3_ and P_97_ were as follows: 3 months (10.29, 22.47), 12 months (12.02, 23.69), 24 months (11.68, 22.20), and 60 months (11.28, 20.20). For girls, the corresponding values were (9.97, 22.27), (11.35, 23.10), (11.09, 21.78), and (10.93, 20.40) (Figure 4 and Table 3a,b).

Notably, the BMI values in the new curves were lower for both sexes compared to WHO standards [22], with differences of 1.59 kg/m^2^ for boys and 1.43 kg/m^2^ for girls at 3 months. Throughout the study, boys lagged by 0.33 at 60 months, while girls were behind by 0.54, all falling below WHO standards (Table 4).

## 4. Discussion

This study explores growth patterns in children aged 3 to 60 months in Punjab, Pakistan, revealing lower weight and shorter length/height as compared to WHO standards, suggesting regional factors like nutritional practices, socioeconomic conditions, and environmental influence on growth. The study suggested that local growth references may accurately reflect the health and nutritional status of children in Punjab, which highlighted the need for context-specific references to better assess and monitor the child’s growth.

Finding anthropological research on children, particularly infants aged 3 to 60 months, living in Punjab, Pakistan, presents a significant challenge. In light of the pressing issues of childhood obesity and undernourishment in the country [23,24], there is an urgent need for detailed statistics regarding the growth status of Pakistan’s pediatric population, especially from a geographical perspective. In the absence of a national standard, child growth in Pakistan has traditionally been assessed against international standards. Recent research [7,13] highlights notable differences between the growth patterns of Pakistani children and the WHO recommendations. To address this gap, novel references for weight, length/height, and BMI tailored to children aged 3 to 60 months have been carefully established in the province of Punjab. This study is part of a comprehensive nationally representative cohort of healthy children within this age range born in Punjab [14]. Remarkably, this research marks one of the first efforts to establish percentiles for anthropometric variables among children in Punjab, Pakistan, following the normalization of skewness and kurtosis.

Employing the GAMLSS framework, we utilized the BCPE. In the first phase of GAMLSS, we identified distinct optimal sub-models for weight, length/height, and BMI, considering each parameter and sex. We determined the degrees of freedom for each anthropometric variable individually, including median (μ), coefficient of variation (σ), skewness (ν), and kurtosis (τ), utilizing cubic spline smoothing functions for all anthropometric parameters to create smooth curves. Experimenting with varying degrees of freedom, we iteratively refined the curves until achieving the desired outcomes. Following approaches similar to those outlined in previous studies [16,21], percentile curves and references were generated for each variable once the desired results were attained. Upon creating the centile curves for this study, a comparative analysis was conducted against international, national, and WHO standards, revealing significant variations. Notably, the age groups of Punjabi children exhibited considerable differences from WHO references, although the difference was less pronounced when compared to national growth references [7] as opposed to findings from other international studies [5,6] and WHO growth references [22].

When comparing the median percentiles (P_50_) of weight-for-age of Punjabi boys and girls with recently published data [7] and another Asian study [5], the findings are consistent from 3 months to 48 months of age; however, they diverge significantly at 60 months. At this age, the observed difference was 1.13 kg in boys and 1.11 kg in girls, potentially attributed to varying sample sizes. In contrast to WHO standards [22] and Egyptian children [6] between 12 and 60 months, substantial variations were noted within the same age groups. Specifically, at 60 months, Punjabi boys and girls exhibited 2.43 kg and 2.81 kg less weight, respectively, when compared to the WHO 2006 standards [22]. During the initial year of life, the Punjabi (Pakistani) boys were 1.39 kg heavier than the Egyptian boys, and the girls were 1.33 kg heavier than their counterparts. However, beyond the first year, the Egyptian children consistently displayed significantly higher weights compared to the Punjabi-Pakistani children, ranging from 0.62 kg to 1.83 kg in boys and 0.30 kg to 2.51 kg in girls (Table 4). It was observed that Punjabi-Pakistani children demonstrated shorter heights compared to WHO standards [22] across all age groups, particularly when compared to reported heights in both national [7] and international studies [5,6] for those aged 24 months or older. When compared to Indian children, Punjabi boys were 1.69 cm longer, and girls were 2.49 cm longer at 6 months of age. However, between the ages of 24 and 60 months, Indian boys were taller, with variations ranging from 0.32 to 4.88 cm, and Indian girls were taller, with variations between 0.96 and 4.75 cm. In contrast to previous findings [5], significant differences between the two countries were observed at 60 months of age, measuring 4.88 cm in boys and 4.75 cm in girls (Table 4). The median BMI percentile reported in our investigation shows significant deviation from previous national [7] and international studies [5,6]. While a limited number of studies [7,8,9,10,11,12,13] have addressed the prevalence of nutritional deficiencies in children [25] and established national growth references for school-age children, there is a conspicuous lack of provincial-level research employing the same ages and methodologies. Understanding the physical and developmental growth of children in this age group is crucial; however, this specific demographic has not received adequate research attention. The implications of this study are significant for both clinical and public health domains. By establishing region-specific growth references, healthcare providers can more accurately assess and monitor pediatric growth, enabling early identification of growth delays and malnutrition. This information is crucial for designing targeted nutritional interventions, promoting exclusive breastfeeding, and improving maternal and child health care services. Future studies incorporating diverse populations and longitudinal data will further enhance the understanding of pediatric growth patterns, allowing for more effective and evidence-based interventions.

### Strengths and Limitations

The strength of this study is evident in the development of the initial smooth trajectories, represented as growth reference charts, for essential anthropometric measures among Punjabi-Pakistani children aged 3 to 60 months. We compared the median percentile values obtained from these smoothed trajectories with WHO standards. Importantly, these percentiles were calculated after adjusting the data for both skewness and kurtosis, employing the contemporary and advanced statistical method of BCPE with GAMLSS, applied to both boys and girls in the province of Punjab, Pakistan.

However, a primary limitation of our study is its exclusive focus on the province of Punjab, and it did not delve into the remaining provinces. The sample could not accurately reflect the diversity of the entire population because it was selected from a particular region. Future research should acknowledge the potential existence of distinct variables influencing growth in children from different provinces. Another drawback is the cross-sectional nature of the data, offering insights into children’s growth status at a specific moment rather than indicating trends over time. Therefore, we recommend future longitudinal research to provide a more comprehensive understanding of population growth trends. The cross-sectional approach to this study restricts our ability to make inferences regarding the long-term growth trajectories of individuals. To gain a deeper understanding of the underlying factors influencing development trends in this group, future research should be multicentric and longitudinal, incorporating through evaluation of nutritional intake, socioeconomic status, and access to healthcare facilities.

## 5. Conclusions

It has been concluded that the new growth reference curves for weight-for-age, length/height-for-age, and BMI-for-age of the Punjab-Pakistan revealed significant variation when compared with WHO standards and several global studies. This study provides the first smoothed growth reference percentiles for Punjabi children aged 3 to 60 months using LMS and BCPE models with the GAMLSS framework. The results highlight that Punjabi children tend to have a lower weight and are smaller in height as compared to international standards, emphasizing the need for region-specific growth monitoring. These findings offer valuable benchmarks for assessing pediatric nutritional status and growth trends in Punjab and beyond. Healthcare professionals and policymakers can use these references to improve child health initiatives, including regular screenings, targeted nutrition programs, and preventive interventions. Pediatricians can leverage these percentiles to enhance early diagnosis and management of growth-related concerns. Routine monitoring using these growth references can help identify at-risk children and ensure timely interventions to promote healthy development. Further research should expand to include diverse populations through multicentric studies, employing robust statistical methodologies at regular intervals. Continuous assessment of growth standards is essential to keep pace with evolving health and nutritional trends. By integrating these findings into clinical practice and policy frameworks, a more comprehensive and effective approach to pediatric health and nutrition in Punjab can be achieved. Furthermore, these findings can guide targeted strategies to promote growth and development in Punjab, leading to more effective preventative measures. Future research should adopt a multicentric approach to achieve a comprehensive understanding of pediatric growth patterns.

## Figures and Tables

**Table 1:** (**a**) Percentile (P) values and L, M, and S parameters of weight-for-age of boys (Punjab). (**b**) Percentile (P) values and L, M, and S parameters of weight-for-age of girls (Punjab).

(a)												
Age (Months)	$P_{3}$	$P_{5}$	$P_{15}$	$P_{25}$	$P_{50}$	$P_{75}$	$P_{85}$	$P_{95}$	$P_{97}$	L	M	S
3	3.49	3.68	4.24	4.60	5.38	6.30	6.86	7.91	8.37	−0.04	5.38	0.23
6	4.82	5.05	5.70	6.13	7.05	8.15	8.82	10.11	10.67	−0.22	7.05	0.21
9	5.80	6.04	6.73	7.19	8.18	9.36	10.09	11.50	12.12	−0.36	8.18	0.20
12	6.49	6.75	7.47	7.96	8.99	10.24	11.02	12.53	13.20	−0.47	8.99	0.19
15	6.96	7.23	7.98	8.49	9.57	10.87	11.69	13.28	13.99	−0.52	9.57	0.18
18	7.32	7.60	8.38	8.90	10.03	11.39	12.23	13.89	14.62	−0.53	10.03	0.18
21	7.65	7.94	8.75	9.30	10.46	11.86	12.73	14.43	15.18	−0.51	10.46	0.18
24	7.97	8.26	9.11	9.68	10.88	12.32	13.21	14.94	15.69	−0.47	10.88	0.18
27	8.28	8.59	9.48	10.07	11.32	12.81	13.72	15.47	16.24	−0.42	11.32	0.18
30	8.60	8.93	9.86	10.48	11.78	13.32	14.26	16.04	16.82	−0.37	11.78	0.18
33	8.94	9.29	10.27	10.92	12.27	13.86	14.82	16.65	17.44	−0.31	12.27	0.18
36	9.29	9.66	10.69	11.36	12.78	14.42	15.41	17.27	18.07	−0.26	12.78	0.18
39	9.64	10.02	11.10	11.80	13.27	14.95	15.97	17.86	18.67	−0.20	13.27	0.18
42	9.94	10.34	11.46	12.20	13.71	15.43	16.46	18.38	19.19	−0.14	13.71	0.17
45	10.21	10.63	11.79	12.54	14.10	15.86	16.90	18.82	19.63	−0.08	14.10	0.17
48	10.46	10.89	12.10	12.88	14.47	16.26	17.32	19.25	20.05	−0.01	14.47	0.17
51	10.70	11.15	12.40	13.20	14.83	16.66	17.72	19.65	20.46	0.05	14.83	0.17
54	10.93	11.39	12.69	13.51	15.18	17.03	18.11	20.04	20.84	0.12	15.18	0.17
57	11.15	11.63	12.97	13.82	15.53	17.41	18.49	20.42	21.22	0.19	15.53	0.17
60	11.36	11.86	13.25	14.12	15.87	17.77	18.86	20.80	21.60	0.26	15.87	0.17
(**b**)												
**Age (Months)**	$P_{3}$	$P_{5}$	$P_{15}$	$P_{25}$	$P_{50}$	$P_{75}$	$P_{85}$	$P_{95}$	$P_{97}$	**L**	**M**	**S**
3	3.26	3.44	3.96	4.31	5.07	5.98	6.54	7.63	8.11	−0.14	5.07	0.24
6	4.33	4.54	5.17	5.59	6.48	7.55	8.20	9.47	10.01	−0.17	6.48	0.22
9	5.15	5.39	6.08	6.55	7.53	8.69	9.41	10.77	11.37	−0.20	7.53	0.21
12	5.76	6.03	6.77	7.27	8.33	9.58	10.34	11.80	12.43	−0.22	8.33	0.20
15	6.24	6.52	7.32	7.85	8.98	10.31	11.13	12.69	13.36	−0.23	8.98	0.20
18	6.61	6.91	7.76	8.33	9.52	10.94	11.81	13.46	14.18	−0.23	9.52	0.20
21	6.93	7.24	8.14	8.73	9.99	11.47	12.38	14.11	14.86	−0.22	9.99	0.20
24	7.22	7.55	8.48	9.10	10.40	11.93	12.86	14.63	15.39	−0.20	10.40	0.20
27	7.52	7.86	8.82	9.46	10.80	12.36	13.31	15.10	15.87	−0.17	10.80	0.20
30	7.83	8.18	9.18	9.83	11.21	12.80	13.77	15.58	16.35	−0.14	11.21	0.20
33	8.15	8.52	9.55	10.23	11.65	13.28	14.26	16.09	16.87	−0.11	11.65	0.19
36	8.50	8.89	9.96	10.66	12.12	13.79	14.79	16.64	17.43	−0.07	12.12	0.19
39	8.86	9.26	10.38	11.11	12.61	14.32	15.34	17.23	18.02	−0.03	12.61	0.19
42	9.20	9.62	10.78	11.53	13.08	14.84	15.88	17.79	18.59	0.01	13.08	0.19
45	9.51	9.94	11.15	11.93	13.52	15.31	16.37	18.30	19.11	0.05	13.52	0.19
48	9.80	10.25	11.49	12.29	13.92	15.75	16.81	18.76	19.56	0.09	13.92	0.18
51	10.08	10.54	11.81	12.63	14.30	16.14	17.22	19.16	19.97	0.13	14.30	0.18
54	10.34	10.81	12.12	12.96	14.65	16.52	17.60	19.55	20.35	0.18	14.65	0.18
57	10.61	11.10	12.44	13.30	15.02	16.91	18.00	19.95	20.75	0.22	15.02	0.18
60	10.88	11.38	12.76	13.64	15.39	17.31	18.40	20.36	21.16	0.26	15.39	0.18

**Table 2:** (**a**) Percentile (P) values and L, M, and S parameters of length/height-for-age of boys (Punjab). (**b**) Percentile (P) values and L, M, and S parameters of length/height-for-age of girls (Punjab).

(a)												
Age (Months)	$P_{3}$	$P_{5}$	$P_{15}$	$P_{25}$	$P_{50}$	$P_{75}$	$P_{85}$	$P_{95}$	$P_{97}$	L	M	S
3	52.16	52.98	55.15	56.50	59.15	61.96	63.55	66.35	67.48	−0.37	59.15	0.07
6	57.53	58.38	60.66	62.08	64.89	67.91	69.63	72.69	73.93	−0.64	64.89	0.07
9	61.70	62.58	64.94	66.43	69.39	72.60	74.44	77.74	79.09	−0.88	69.39	0.07
12	64.83	65.74	68.20	69.76	72.88	76.31	78.29	81.87	83.35	−1.09	72.88	0.07
15	67.24	68.19	70.77	72.42	75.73	79.40	81.54	85.45	87.09	−1.25	75.73	0.07
18	69.21	70.20	72.92	74.66	78.18	82.12	84.43	88.70	90.50	−1.37	78.18	0.07
21	70.89	71.92	74.78	76.62	80.35	84.56	87.05	91.67	93.63	−1.44	80.35	0.07
24	72.44	73.53	76.53	78.46	82.40	86.86	89.51	94.45	96.55	−1.45	82.40	0.08
27	74.00	75.14	78.28	80.31	84.45	89.15	91.94	97.15	99.37	−1.41	84.45	0.08
30	75.53	76.73	80.03	82.16	86.50	91.41	94.33	99.76	102.07	−1.33	86.50	0.08
33	77.04	78.29	81.75	83.97	88.49	93.59	96.61	102.20	104.57	−1.22	88.49	0.08
36	78.56	79.87	83.47	85.78	90.46	95.69	98.78	104.45	106.83	−1.08	90.46	0.08
39	80.11	81.48	85.21	87.60	92.39	97.72	100.83	106.50	108.87	−0.92	92.39	0.08
42	81.56	82.98	86.83	89.28	94.16	99.52	102.63	108.24	110.56	−0.74	94.16	0.08
45	82.84	84.30	88.26	90.75	95.69	101.06	104.13	109.63	111.88	−0.54	95.69	0.08
48	84.06	85.57	89.63	92.16	97.15	102.49	105.53	110.90	113.07	−0.33	97.15	0.08
51	85.30	86.86	91.01	93.59	98.62	103.94	106.94	112.18	114.29	−0.11	98.62	0.08
54	86.49	88.10	92.37	95.00	100.08	105.40	108.36	113.51	115.56	0.10	100.08	0.08
57	87.66	89.33	93.73	96.42	101.57	106.91	109.86	114.94	116.95	0.30	101.57	0.08
60	88.84	90.57	95.12	97.88	103.12	108.50	111.44	116.47	118.45	0.51	103.12	0.08
(**b**)												
**Age (Months)**	$P_{3}$	$P_{5}$	$P_{15}$	$P_{25}$	$P_{50}$	$P_{75}$	$P_{85}$	$P_{95}$	$P_{97}$	**L**	**M**	**S**
3	51.01	51.79	53.92	55.28	58.03	61.10	62.90	66.20	67.59	−1.20	58.03	0.07
6	56.73	57.53	59.72	61.13	64.00	67.24	69.17	72.75	74.27	−1.58	64.00	0.07
9	60.34	61.16	63.45	64.92	67.96	71.43	73.51	77.44	79.13	−1.83	67.96	0.07
12	63.06	63.93	66.33	67.90	71.14	74.89	77.17	81.52	83.42	−2.00	71.14	0.07
15	65.46	66.38	68.93	70.61	74.09	78.16	80.65	85.45	87.57	−2.08	74.09	0.08
18	67.57	68.54	71.26	73.04	76.77	81.15	83.85	89.07	91.39	−2.08	76.77	0.08
21	69.39	70.42	73.29	75.18	79.13	83.78	86.65	92.20	94.67	−2.02	79.13	0.08
24	71.13	72.21	75.21	77.18	81.29	86.10	89.05	94.75	97.25	−1.90	81.29	0.08
27	72.77	73.90	77.02	79.06	83.28	88.18	91.16	96.85	99.33	−1.73	83.28	0.08
30	74.27	75.44	78.69	80.79	85.13	90.11	93.10	98.75	101.19	−1.54	85.13	0.08
33	75.68	76.90	80.28	82.46	86.92	91.98	94.99	100.59	102.98	−1.32	86.92	0.08
36	77.13	78.41	81.94	84.21	88.79	93.92	96.94	102.49	104.83	−1.08	88.79	0.08
39	78.60	79.96	83.65	86.00	90.72	95.92	98.95	104.44	106.72	−0.82	90.72	0.08
42	80.00	81.42	85.30	87.74	92.59	97.87	100.90	106.33	108.56	−0.55	92.59	0.08
45	81.29	82.80	86.85	89.38	94.35	99.68	102.71	108.05	110.22	−0.28	94.35	0.08
48	82.52	84.10	88.33	90.94	96.02	101.38	104.37	109.61	111.72	0.00	96.02	0.08
51	83.69	85.35	89.74	92.43	97.59	102.94	105.91	111.02	113.05	0.29	97.59	0.08
54	84.84	86.58	91.12	93.87	99.09	104.43	107.35	112.33	114.29	0.56	99.09	0.08
57	86.00	87.81	92.49	95.30	100.57	105.89	108.76	113.62	115.51	0.83	100.57	0.08
60	87.15	89.03	93.87	96.73	102.05	107.34	110.17	114.92	116.75	1.09	102.05	0.08

**Table 3:** (**a**) Percentile (P) values and L, M, and S parameters of length/height-for-age of boys (Punjab). (**b**) Percentile (P) values and L, M, and S parameters of BMI-for-age of girls (Punjab).

(a)												
Age (Months)	$P_{3}$	$P_{5}$	$P_{15}$	$P_{25}$	$P_{50}$	$P_{75}$	$P_{85}$	$P_{95}$	$P_{97}$	L	M	S
3	10.29	10.82	12.32	13.29	15.31	17.59	18.94	21.43	22.47	0.09	15.31	0.21
6	11.15	11.68	13.18	14.15	16.15	18.42	19.76	22.23	23.26	0.04	16.15	0.20
9	11.76	12.28	13.76	14.72	16.69	18.92	20.24	22.67	23.69	0.00	16.69	0.19
12	12.02	12.54	13.98	14.91	16.84	19.02	20.31	22.69	23.69	−0.04	16.84	0.18
15	12.01	12.51	13.91	14.83	16.70	18.83	20.09	22.42	23.40	−0.07	16.70	0.18
18	11.89	12.38	13.74	14.63	16.45	18.52	19.75	22.03	22.99	−0.10	16.45	0.18
21	11.78	12.25	13.57	14.43	16.20	18.23	19.43	21.65	22.59	−0.12	16.20	0.17
24	11.68	12.14	13.42	14.26	15.98	17.95	19.12	21.29	22.20	−0.15	15.98	0.17
27	11.60	12.05	13.29	14.10	15.78	17.69	18.83	20.95	21.84	−0.17	15.78	0.17
30	11.54	11.98	13.19	13.98	15.62	17.49	18.60	20.67	21.55	−0.20	15.62	0.17
33	11.53	11.96	13.15	13.93	15.54	17.38	18.49	20.54	21.41	−0.23	15.54	0.16
36	11.53	11.95	13.14	13.91	15.51	17.34	18.44	20.49	21.36	−0.26	15.51	0.16
39	11.50	11.91	13.08	13.85	15.44	17.27	18.36	20.42	21.30	−0.28	15.44	0.16
42	11.43	11.84	13.00	13.75	15.33	17.15	18.25	20.30	21.18	−0.31	15.33	0.16
45	11.37	11.78	12.92	13.68	15.24	17.06	18.15	20.21	21.09	−0.33	15.24	0.16
48	11.35	11.76	12.89	13.64	15.19	17.00	18.09	20.14	21.02	−0.35	15.19	0.16
51	11.35	11.75	12.87	13.60	15.14	16.92	17.99	20.01	20.88	−0.36	15.14	0.16
54	11.34	11.73	12.83	13.56	15.06	16.80	17.85	19.83	20.68	−0.36	15.06	0.16
57	11.32	11.70	12.79	13.50	14.97	16.67	17.70	19.62	20.45	−0.36	14.97	0.16
60	11.28	11.66	12.72	13.42	14.87	16.53	17.53	19.40	20.20	−0.36	14.87	0.15
(**b**)												
**Age (Months)**	$P_{3}$	$P_{5}$	$P_{15}$	$P_{25}$	$P_{50}$	$P_{75}$	$P_{85}$	$P_{95}$	$P_{97}$	**L**	**M**	**S**
3	9.97	10.49	11.98	12.95	14.97	17.28	18.65	21.20	22.27	0.06	14.97	0.21
6	10.84	11.38	12.90	13.88	15.90	18.18	19.53	22.00	23.03	0.09	15.90	0.20
9	11.23	11.77	13.28	14.26	16.25	18.50	19.81	22.21	23.21	0.10	16.25	0.19
12	11.35	11.89	13.37	14.33	16.30	18.49	19.78	22.13	23.10	0.10	16.30	0.19
15	11.32	11.85	13.31	14.25	16.17	18.33	19.59	21.89	22.85	0.09	16.17	0.19
18	11.24	11.76	13.18	14.10	15.98	18.09	19.32	21.58	22.52	0.07	15.98	0.18
21	11.15	11.65	13.03	13.93	15.76	17.81	19.02	21.23	22.15	0.05	15.76	0.18
24	11.09	11.57	12.91	13.78	15.56	17.56	18.73	20.89	21.78	0.02	15.56	0.18
27	11.06	11.53	12.84	13.69	15.42	17.38	18.53	20.64	21.52	−0.01	15.42	0.18
30	11.06	11.52	12.80	13.63	15.33	17.26	18.39	20.48	21.35	−0.03	15.33	0.17
33	11.07	11.52	12.78	13.60	15.28	17.19	18.32	20.39	21.26	−0.06	15.28	0.17
36	11.08	11.53	12.78	13.59	15.25	17.15	18.27	20.33	21.20	−0.09	15.25	0.17
39	11.10	11.54	12.77	13.57	15.23	17.11	18.22	20.28	21.15	−0.12	15.23	0.17
42	11.09	11.52	12.74	13.54	15.18	17.05	18.16	20.22	21.09	−0.14	15.18	0.17
45	11.06	11.49	12.70	13.49	15.12	16.98	18.08	20.14	21.00	−0.16	15.12	0.17
48	11.03	11.46	12.65	13.43	15.05	16.89	17.99	20.03	20.90	−0.18	15.05	0.17
51	11.00	11.42	12.60	13.37	14.97	16.79	17.88	19.91	20.77	−0.20	14.97	0.17
54	10.97	11.39	12.55	13.31	14.89	16.69	17.77	19.78	20.64	−0.21	14.89	0.17
57	10.95	11.36	12.51	13.26	14.82	16.61	17.67	19.67	20.51	−0.23	14.82	0.17
60	10.93	11.34	12.47	13.22	14.76	16.53	17.58	19.56	20.40	−0.24	14.76	0.17

**Table 4:** Comparison of median percentiles of the present study with WHO 2006, national, and international studies.

Age (Months)	Punjab Pakistan Present Study		WHO 2006 [22]		Pakistani 2022 [7]		India 2019 [5]		Egyptian 2021 [6]	
	Boys	Girls	Boys	Girls	Boys	Girls	Boys	Girls	Boys	Girls
	Weight (kg)									
	$P_{50}$	$P_{50}$	$P_{50}$	$P_{50}$	$P_{50}$	$P_{50}$	$P_{50}$	$P_{50}$	$P_{50}$	$P_{50}$
3	5.38	5.07	6.40	5.80	5.36	5.09	-----	-----	------	-----
6	7.05	6.48	7.90	7.30	6.9	6.5	6.9	6.8	------	-----
12	8.99	8.33	9.6	8.9	8.8	8.2	8.9	8.9	7.6	7.0
24	10.88	10.40	12.2	11.5	10.8	10.2	10.7	10.7	11.5	10.7
36	12.78	12.12	14.3	13.9	12.5	11.9	12.7	12.4	13.8	13.4
48	14.47	13.92	16.3	16.1	14.1	13.6	14.8	14.4	15.7	15.9
60	15.87	15.39	18.3	18.2	15.5	15.0	17.0	16.5	17.7	17.9
	**Length/height (cm)**									
3	59.15	58.03	61.4	59.8	58.88	57.99	-----	-----	-----	------
6	64.89	64.00	67.6	65.7	65.3	63.7	63.2	62.4	------	------
12	72.88	71.14	75.7	74.0	72.4	70.9	73.2	72.1	65.2	63.8
24	82.40	81.29	87.1	86.4	81.9	80.6	85.3	84.6	81.9	80.2
36	90.46	88.79	96.1	95.1	89.9	88.1	92.9	92.6	91.5	90.2
48	97.15	96.02	103.3	102.7	96.4	95.3	100.6	99.7	99.5	98.6
60	103.12	102.05	110.0	109.4	102.1	100.7	108.0	106.8	106.3	105.7
	**BMI (kg/m^2^)**									
3	15.31	14.97	16.9	16.4	15.55	15.23	-----	------	------	------
6	16.15	15.90	17.3	16.9	16.4	16.0	14.3	14.3	------	------
12	16.84	16.30	16.8	16.4	16.8	16.2	14.9	14.8	17.50	17.3
24	15.98	15.56	15.7	15.4	16.0	15.6	15.2	14.9	17.31	16.9
36	15.51	15.25	15.8	15.4	15.4	15.3	15.0	14.7	16.58	16.3
48	15.19	15.05	15.3	15.3	15.1	14.9	14.8	14.5	16.01	15.8
60	14.87	14.76	15.2	15.3	14.9	14.7	14.7	14.5	15.74	15.6

## Data Availability

Data presented in this study are available on request from the corresponding author. The data are not publicly available due to personal health information.

## Abbreviations

The following abbreviations are used in this manuscript:

MDPI	Multidisciplinary Digital Publishing Institute
DOAJ	Directory of open access journals
TLA	Three letter acronym
LD	Linear dichroism

## Appendix A

**Table A1:** Theoretical percentiles and empirical percentiles of the fitted model of Punjab.

Theoretical Percentiles	Empirical Percentiles			Empirical Percentiles		
	Boys			Girls		
	Weight (kg)	Height (cm)	BMI (kg/m^2^)	Weight (kg)	Height (cm)	BMI (kg/m^2^)
3	3.47	3.38	2.92	3.35	3.40	3.67
5	4.89	5.39	4.77	5.15	5.37	5.31
15	15.23	15.02	14.38	14.89	14.87	14.49
25	24.93	24.52	24.52	24.59	24.64	24.27
50	49.87	49.59	50.54	50.26	49.98	50.76
75	74.84	75.86	75.06	75.48	75.88	76.21
85	84.69	85.64	85.77	84.94	85.40	85.85
95	95.87	94.23	95.04	94.53	94.16	94.63
97	97.75	96.22	96.99	96.71	96.57	96.61

**Table A2:** Descriptive statistics of boys’ (Punjab) weight, height, and BMI.

Age (Months)	n	Weight (kg)				Height (cm)				BMI (kg/m^2^)			
		Mean ± SD	Median	Skew	Kurt	Mean ± SD	Median	Skew	Kurt	Mean ± SD	Median	Skew	Kurt
3	487	5.04 ± 1.14	5.00	0.84	4.36	57.51 ± 4.76	57.70	−0.03	2.28	15.11 ± 3.34	14.72	0.56	3.69
6	506	6.99 ± 1.57	6.80	0.85	4.29	65.06 ± 4.65	65.00	0.26	3.41	16.42 ± 3.22	15.98	0.75	3.59
9	528	8.20 ± 1.67	8.00	0.62	3.82	68.84 ± 3.86	68.80	0.14	3.04	17.23 ± 3.23	16.79	0.57	3.57
12	531	9.17 ± 1.82	8.90	1.24	4.88	72.88 ± 4.92	72.40	0.65	3.62	17.18 ± 3.08	16.60	0.80	3.68
15	549	9.75 ± 1.89	9.50	1.06	4.61	75.38 ± 5.35	75.00	0.55	3.45	17.01 ± 3.06	16.49	0.70	3.70
18	551	10.14 ± 2.08	9.80	0.96	3.92	78.32 ± 5.86	77.6	0.46	2.79	16.52 ± 3.03	16.19	0.51	3.49
21	522	10.72 ± 1.95	10.45	0.69	3.25	80.74 ± 6.29	80.1	0.83	4.27	16.53 ± 2.91	16.27	0.67	4.04
24	447	11.11 ± 2.13	10.80	0.87	3.78	82.18 ± 6.75	81.4	0.74	3.83	16.45 ± 2.94	16.10	0.99	4.36
27	591	11.48 ± 2.10	11.20	0.78	3.67	84.50 ± 6.44	83.80	0.69	4.04	16.13 ± 2.86	15.78	0.92	4.66
30	550	11.84 ± 2.12	11.70	0.60	3.98	86.74 ± 7.04	85.80	0.71	3.62	15.78 ± 2.57	15.54	0.54	4.36
33	585	12.46 ± 2.27	12.30	0.82	4.08	89.28 ± 7.65	88.50	0.62	3.68	15.70 ± 2.65	15.37	0.83	4.59
36	570	12.84 ± 2.46	12.50	0.79	3.76	90.02 ± 7.41	89.50	0.37	2.99	15.88 ± 2.70	15.48	0.96	4.60
39	624	13.52 ± 2.28	13.30	0.48	3.27	92.70 ± 7.61	92.20	0.34	3.38	15.81 ± 2.62	15.29	0.81	4.22
42	563	14.11 ± 2.52	13.70	0.72	3.55	95.33 ± 7.91	94.70	0.65	3.69	15.58 ± 2.67	15.07	1.13	5.00
45	581	14.21 ± 2.51	14.00	0.72	3.71	95.92 ± 7.81	95.70	0.58	3.72	15.48 ± 2.54	15.07	0.83	4.04
48	610	14.44 ± 2.54	14.30	0.50	3.48	96.74 ± 7.68	96.60	0.27	3.25	15.47 ± 2.72	15.15	0.89	4.95
51	514	15.15 ± 2.73	14.90	0.47	2.99	99.03 ± 7.80	99.20	0.22	3.81	15.52 ± 2.74	15.04	1.11	4.55
54	529	15.25 ± 2.56	14.90	0.53	3.04	99.89 ± 7.30	100.20	0.25	3.56	15.32 ± 2.52	14.81	1.10	5.00
57	533	15.62 ± 2.66	15.30	0.33	2.86	101.33 ± 7.71	101.10	0.08	3.11	15.27 ± 2.46	14.92	0.77	4.14
60	360	15.99 ± 2.70	15.80	0.14	2.50	103.08 ± 8.19	102.35	0.23	2.96	15.11 ± 2.35	14.82	0.63	3.97
Total	10,731												

**Table A3:** Descriptive statistics of girls’ (Punjab) weight, height, and BMI.

Age (Months)	n	Weight (kg)				Height (cm)				BMI (kg/m^2^)			
		Mean ± SD	Median	Skew	Kurt	Mean ± SD	Median	Skew	Kurt	Mean ± SD	Median	Skew	Kurt
3	484	4.88 ± 1.43	4.60	1.07	4.42	56.71 ± 5.11	56.40	0.63	4.50	15.09 ± 3.43	14.46	0.75	3.74
6	474	6.44 ± 1.49	6.40	0.65	4.23	63.54 ± 4.60	63.00	0.72	4.27	15.91 ± 3.28	15.54	0.51	3.43
9	507	7.59 ± 1.53	7.50	0.73	3.96	67.78 ± 4.87	67.60	0.28	3.16	16.55 ± 3.23	15.98	0.78	3.83
12	470	8.41 ± 1.75	8.10	1.16	5.22	71.01 ± 5.59	70.10	1.22	5.84	16.69 ± 3.14	16.27	0.56	3.80
15	540	9.08 ± 1.90	8.80	0.97	4.29	74.38 ± 6.08	73.50	0.91	4.13	16.41 ± 3.08	16.13	0.66	3.78
18	481	9.60 ± 1.91	9.40	0.73	3.77	76.87 ± 5.75	76.50	0.71	3.73	16.22 ± 2.91	15.93	0.56	3.96
21	519	10.42 ± 2.54	9.80	0.88	3.31	79.89 ± 7.45	78.60	0.72	3.36	16.22 ± 3.25	15.69	0.72	3.59
24	450	10.47 ± 2.09	10.20	0.69	3.61	81.93 ± 7.29	80.70	0.84	3.65	15.65 ± 2.69	15.51	0.46	3.68
27	559	10.98 ± 2.20	10.70	0.73	3.72	83.76 ± 7.06	83.20	0.71	4.09	15.68 ± 2.79	15.38	0.81	4.76
30	556	11.29 ± 2.11	10.90	0.63	3.99	85.27 ± 6.72	84.60	0.68	3.89	15.58 ± 2.75	15.35	0.71	4.76
33	531	11.91 ± 2.47	11.60	0.93	4.45	87.49 ± 7.74	86.40	0.86	4.22	15.58 ± 2.65	15.42	0.95	5.59
36	538	12.09 ± 2.47	11.90	0.66	3.79	88.75 ± 7.42	88.30	0.49	3.56	15.42 ± 2.99	15.14	0.74	4.49
39	519	12.64 ± 2.14	12.40	0.68	3.88	90.53 ± 6.74	90.10	0.47	3.75	15.48 ± 2.43	15.17	1.08	5.62
42	551	13.45 ± 2.60	13.00	0.63	3.22	93.32 ± 7.95	92.90	0.47	3.44	15.52 ± 2.81	15.10	0.98	4.22
45	528	13.67 ± 2.62	13.30	0.60	3.26	94.35 ± 7.72	94.10	0.36	3.33	15.40 ± 2.68	14.98	0.92	4.39
48	523	14.15 ± 2.71	13.80	0.42	3.26	95.88 ± 7.96	95.80	0.31	3.40	15.47 ± 2.88	14.96	0.78	4.24
51	502	14.45 ± 2.55	14.20	0.51	3.32	98.11 ± 8.11	98.10	0.28	3.33	15.09 ± 2.56	14.58	0.82	4.34
54	504	14.77 ± 2.60	14.60	0.42	3.19	98.75 ± 7.64	99.15	−0.08	3.22	15.21 ± 2.59	14.84	0.96	4.52
57	498	14.91 ± 2.74	14.60	0.45	2.99	100.06 ± 7.89	100.00	0.06	3.26	14.97 ± 2.69	14.49	0.87	4.96
60	380	15.63 ± 2.71	15.40	0.29	2.84	101.87 ± 7.65	102.05	−0.01	3.05	15.11 ± 2.46	14.66	1.06	5.40
Total	10,114												
